# Interteam Cooperation and Competition and Boundary Activities: The Cross-Level Mediation of Team Goal Orientations

**DOI:** 10.3390/ijerph16152738

**Published:** 2019-07-31

**Authors:** Yuhyung Shin, Mihee Kim, Won-Moo Hur

**Affiliations:** 1 School of Business, Hanyang University, 17 Haengdang-dong, Seongdong-gu, Seoul 133-791, Korea; 2 College of Business Administration, Inha University, 100 Inha-ro, Michuhol-gu, Incheon 22212, Korea

**Keywords:** interteam cooperation, interteam competition, boundary spanning, boundary reinforcement, boundary buffering, team learning goal orientation, team performance-prove goal orientation, team performance-avoid goal orientation

## Abstract

Drawing on Dragoni’s cross-level model of state goal orientation, this research aims to examine the cross-level mediating effect of team goal orientation on the relationships between interteam cooperation and competition and three forms of boundary activities. Study 1 tested the proposed mediating relationships by collecting survey data from 249 members of 45 South Korean work teams. Additionally, we conducted a two-wave longitudinal study (Study 2) on 188 undergraduate students to replicate the relationships between three types of team goal orientation and their relevant forms of boundary activities. In Study 1, we found positive associations between interteam cooperation and team learning goal orientation, and between interteam competition and team performance-prove and performance-avoid goal orientations. Team learning and performance-prove goal orientations were positively related to boundary spanning and reinforcement. As predicted, team learning goal orientation had a stronger relationship with boundary spanning than team performance-prove goal orientation, whereas team performance-prove goal orientation had a stronger relationship with boundary reinforcement than team learning goal orientation. While team learning goal orientation mediated the relationship between interteam cooperation and boundary spanning and reinforcement, team performance-prove goal orientation mediated the relationship between interteam competition and boundary spanning and reinforcement. The results of Study 2 demonstrated the positive lagged effects of team performance-prove goal orientation on boundary reinforcement and of team performance-avoid goal orientation on boundary buffering.

## 1. Introduction

The increasing decentralization of organizational structures and the broad spread of network organizations, virtual teams, and cross-functional teams are blurring work team boundaries and causing the ability to effectively coordinate with external parties to be a key determinant of team performance and innovation [1,2,3,4,5]. This trend triggered much research on boundary activities, which refer to activities that establish and maintain team boundaries and manage interactions across those boundaries [3]. The majority of boundary work research focused on team boundary activities [6]. This stream of research identified team structure and composition [7] and task characteristics [8,9] as predictors of team boundary activities. However, relatively little knowledge is available with respect to team-level factors affecting individual members’ boundary activities. This is a critical omission in light of the mounting evidence for between-individual variance in boundary activities (e.g., References [4,10]). Because team members play differential roles in their team’s boundary activities, they tend to display certain variations in boundary activities [4]. For this reason, researchers constantly called for multilevel research on boundary activities to understand boundary activities that unfold at different levels of organizations [4]. 

Unfortunately, Marrone et al.’s [4] research is the only empirical research that revealed the multilevel forces influencing individual-level boundary activities. They found that individual boundary-spanning behavior is affected by team-level (i.e., external focus and consensus on external focus) and individual-level predictors (i.e., boundary-spanning role and boundary-spanning self-efficacy). While Marrone et al.’s [4] research provides initial, meaningful insights into the critical role of multilevel factors in predicting individual boundary-spanning behavior, we seek to extend their research in three ways. Firstly, as suggested by the boundary management literature, there are forms of boundary activities other than boundary-spanning behavior. That is, in some situations, team members engage in boundary-spanning behavior such as reaching out to external constituents and developing partnerships with them. However, in other circumstances, team members choose to engage in more internally oriented behavior such as reinforcing and buffering team boundaries [1,3]. The boundary management literature highlights that all three forms of boundary activities (i.e., boundary spanning, reinforcement, and buffering) differentially contribute to team functioning [3,6]. Therefore, for a comprehensive understanding of individual team members’ boundary activities, it is necessary to explore antecedents affecting the three forms of boundary activities. 

Secondly, Marrone et al. [4] attended to team-level characteristics such as external focus and consensus on external focus as precursors of individual boundary-spanning behavior. However, because boundary activities involve interteam interactions, interteam dynamics or relations need to be considered a key antecedent of individual boundary activities. Building on the construal process framework of intergroup relations [11], which posits that team members’ perceptions of intergroup cooperation and competition determine their intergroup behaviors, we argue that team members’ perceptions of interteam cooperation and competition serve as pivotal contextual factors influencing their boundary activities after controlling for their individual differences. Thus, the aim of our study is to examine how interteam cooperation and competition affect different boundary activities at the individual level. 

Thirdly, we complement Marrone et al.’s [4] research by uncovering the intermediary processes linking interteam cooperation and competition and different forms of boundary activities. Although interteam relations or interdependence was proposed to influence boundary activities [5,8,12], mediating mechanisms via which interteam relations or interdependence instigate boundary activities are still unknown. Even Marrone et al.’s [4] research did not explore the mediating process between a team’s external focus and individual boundary-spanning activities. To bridge this gap, we attempt a cross-level analysis of mediating processes in which interteam cooperation and competition lead to team members’ boundary activities. Drawing on Dragoni’s [13] cross-level model of state goal orientation, we propose team goal orientation as a central cross-level mechanism that translates the effect of interteam cooperation and competition on individual-level boundary activities. Team goal orientation is defined as the team members’ shared perceptions of the goals pursued by the team [13,14,15,16,17] and is categorized into learning, performance-prove, and performance-avoid goal orientations [17]. In this study, interteam cooperation and competition are hypothesized to affect different forms of boundary activities by shaping their relevant types of team goal orientation within a team. 

The examination of team goal orientation as a mediator between interteam cooperation and competition and boundary activities advances the existing boundary work literature. So far, how interteam relations affect boundary activities and what team processes intervene in such a relationship remain a black box in the boundary management literature. Thus, elucidating disparate mediating processes underlying different boundary activities can provide a sophisticated understanding of boundary activities. In particular, compared with research that examines interteam goal interdependence as an antecedent or a moderator of boundary activities [5,8], relatively little attention was devoted to the role of team goals in promoting boundary activities. Given that boundary activities are behaviors geared toward the achievement of task-related goals [1,18], it is necessary to investigate the role that team goal orientation plays in team members’ boundary activities. Therefore, the objective of this study is to examine the cross-level mediating effect of different team goal orientations on the relationships between interteam cooperation and competition and distinct forms of individual boundary activities. 

## 2. Theoretical Background

Boundary activities encompass team members’ boundary management behaviors targeted at counterpart teams in the same organization, the upper management, and external constituents (e.g., customers, competitors, and government) [8,9]. In line with prior research (e.g., References [5,8]), we limit the scope of boundary activities in this study to activities between a target team and its counterpart teams within the same organization. Adopting Faraj and Yan’s [3] typology, we classify boundary activities into boundary spanning, reinforcement, and buffering. *Boundary spanning* is defined as team members’ actions to reach out to their counterpart teams to acquire important resources and support. Boundary spanning corresponds to ambassador, task coordinator, and scout activities in Ancona and Caldwell’s [1] pioneering study on boundary work. This includes activities such as bargaining and negotiation, contracting and cooptation, and alliance and coalition building [3,19]. *Boundary reinforcement* pertains to activities that create and maintain the team boundary by enhancing member awareness of boundaries and team identity [3,19]. Creating a clear sense of a common task and a collective identity is an example of boundary reinforcement. Although boundary reinforcement was not identified as a specific form of boundary activity by Ancona and Caldwell [1], Yan and Louis [19] introduced “bringing-up boundaries” as inward-facing boundary activities that help the team deal with external constituents. Extending Yan and Louis’s work, Faraj and Yan [3] coined the term boundary reinforcement to represent bringing-up boundaries behavior. *Boundary buffering* refers to a team’s self-protective strategy of closing itself off from exposure to external uncertainties and disturbances [3,19]. Similar to guard activities in Ancona and Caldwell’s [1] research, boundary buffering is exemplified by activities such as limiting interactions with external constituents, preventing the inflow of information and resources from external agents, and controlling the outflow of critical information from the team.

According to the boundary management literature, although internal (i.e., boundary reinforcement and buffering) and external (i.e., boundary spanning) boundary activities are seemingly contradictory, they are not mutually exclusive, as indicated by a positive correlation between boundary-spanning and boundary-strengthening activities [20,21]. It was found that team members alternate between internal and external boundary activities depending on task requirements and the phase of task development [6]. For instance, Sawyer et al.’s [22] research on software development teams showed that team members display more boundary-spanning behavior in the requirement definition phase, whereas boundary reinforcement increases in the software development stage. Dey and Ganesh’s [6], in their review of boundary work research, concluded that a high level of team performance and effectiveness can be found in teams that are capable of balancing between boundary-spanning and boundary-strengthening activities. These findings suggest that team members engage in more than one form of boundary activities and that all three forms of boundary activities are necessary for optimal team functioning.

Drawing on the construal process framework of intergroup relations [11], we propose that boundary activities are driven by team members’ perceptions of whether their team is in a cooperative or competitive stance with other teams. The construal process framework of intergroup relations conceptualizes interteam cooperation and competition based on team members’ perceptions of the team’s current situation [11]. This is due to the reasoning that individuals’ subjective perception and interpretation of a potentially cooperative or competitive situation more strongly affect their behavior than the actual situation itself. Adopting Maxwell-Smith et al.’s [11] conceptualization, we define interteam competition as the perception that a group and another group(s) are striving to acquire a reward or desired outcome at each other’s expense. In contrast, interteam cooperation refers to the perception that the goal achievement of one team increases the ability of other teams to obtain desired outcomes (e.g., goals, rewards) [23,24]. For instance, a human resource development (HRD) team is likely to exhibit a high degree of interteam cooperation because its performance is evaluated based on their client teams’ satisfaction with the training programs offered by the HRD team. On the contrary, a high degree of interteam competition can be found in a bank branch that competes with other branches to win the incentive for the best-performing branch. As work-related interactions frequently occur at the work-unit level [25], collective experience among team members form converged perceptions of the extent to which their team is cooperative or competitive with other teams [26,27,28]. Social information processing theory [29] postulates that repeated exposure to cooperative or competitive situations makes team members develop shared perceptions and interpretations of those situations. Through frequent interactions and communication within the team, team members come to hold shared perceptions of the degrees of cooperation and competition between their team and counterpart teams [30], which constitute interteam cooperation and competition climates. Therefore, over time, team members develop relatively homogeneous perceptions of interteam cooperation and competition, which affect their boundary activities [11].

The construal process framework of intergroup relations maintains that whether individuals perceive their goals as being positively or negatively related to other teams determines how they interact with one another [11,31]. Based on this argument, it is speculated that the relationship of a team’s goals to those of other teams can have important implications for boundary activities between the teams. Although the construal process framework itself does not propose a mediating mechanism via which interteam cooperation and competition affect individual boundary activities, we integrate this framework with Dragoni’s [13] cross-level model of state goal orientation. Dragoni’s model assumes a cross-level causal relationship in which work group climates influence group members’ goal orientations, which in turn affect their behavior. According to this model, team climate signals what is desired and emphasized by the team, thereby motivating team members to adopt a goal orientation that pursues what is desired and emphasized by the team [13]. Based on this theorizing, we propose that interteam cooperation and competition climates precede team members’ choice of goal orientation. For instance, team members holding a cooperative climate perceive counterpart teams as resources for learning and development. As a result, they tend to choose a learning goal orientation as their primary mode of goal orientation. On the other hand, members of teams with a competitive climate regard counterpart teams as competitors, which facilitates a performance goal orientation. Dragoni’s model further claims that team members’ goal orientations determine their resultant behavior. Similarly, well established in the goal orientation literature is that goal orientations are drivers of behavior and performance at both individual and team levels ([32]), which implies that goal orientations precede individuals’ boundary activities. Therefore, we propose a cross-level mediation model in which interteam cooperation and competition climates predict team members’ boundary activities through team goal orientations. Drawing on Dragoni’s model, we identify team learning, performance-prove, and performance-avoid goal orientations as mediators in our model. 

**Figure 1:**
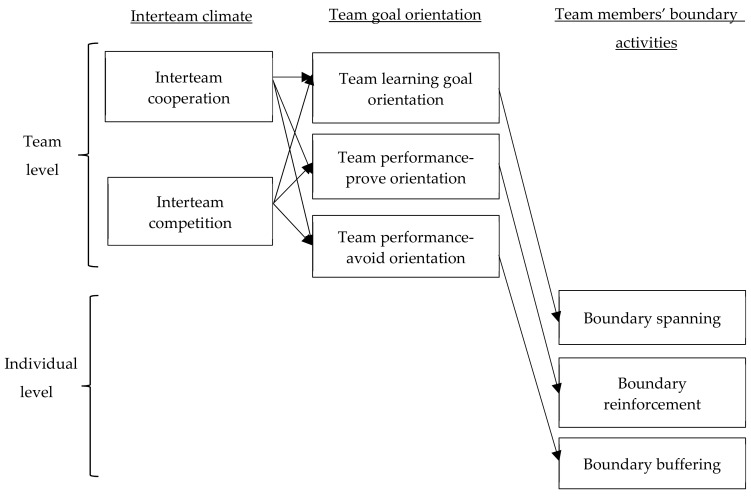
Proposed research model.

Team learning goal orientation is a collective state in which team members perceive their team as pursuing the development of the members’ skills, knowledge, and competence [13,17]. Team performance-prove goal orientation is a collective state in which team members perceive their team as interested in proving its competence over others and attaining favorable assessment [13,17]. Team performance-avoid goal orientation is a collective state in which team members perceive their team as striving to avoid negative assessment. According to Dragoni’s [13] model, different climates affect individual performance and work behavior by shaping relevant forms of goal orientation. Drawing on this logic, we postulate that interteam cooperation leads to team members’ boundary spanning by eliciting a learning goal orientation within the team. On the other hand, interteam competition is expected to affect team members’ boundary reinforcement by creating a team performance-prove goal orientation. Interteam competition is also posited to be linked to team members’ boundary buffering through the intervening mechanism of team performance-avoid goal orientation. The overall structure of our mediation model is in line with the input–mediator–outcome (IMO) model wherein team and organizational contexts (e.g., climate, interteam relations) affect team member outcomes through team emergent states [31]. The team goal orientation literature identified team goal orientation as a crucial team process variable (e.g., References [32,33]). Thus, we propose a mediation model in which interteam cooperation and competition affect different boundary activities through the three types of team goal orientation. The proposed model is illustrated in Figure 1 and is explained in detail in the following sections.

## 3. Hypothesis Development

### 3.1. Team-Level Relationship between Interteam Cooperation and Competition and Team Goal Orientation

The team goal orientation literature theorizes that the climate shared at the team-level determines team goal orientation [17]. When interteam cooperation climate is prevalent within a team, team members perceive that their attainment of desired outcomes increases other teams’ ability to obtain rewards or goals [24,25]. Such a cooperative climate facilitates the exchange and coordination of resources, as well as communication among teams [8,34]. Increased interteam coordination and communication enhance knowledge sharing and information exchange among teams [9,34], which shapes a learning goal orientation within the team. 

However, interteam cooperation is predicted to decrease team performance-prove and performance-avoid goal orientations. This relationship is well explained by social identity theory [35,36]. One tenet of social identity theory is that interteam relations strongly influence the identities of team members. More specifically, when there are fierce competitions between teams, team members’ identification with their team strengthens, whereas interteam cooperation causes team members to experience a dual identity where they identify with both the team and the organization [37]. Therefore, team members tend to have a weaker team identity and to identify themselves with the organization when there is a cooperative climate among teams [12]. In this case, teams are more likely to collaborate across the blurred interteam boundaries [38,39] and, therefore, outperforming other teams becomes less important. Thus, interteam cooperation dilutes team members’ concern for performance evaluation and directs team members’ focus toward long-term growth and learning. Therefore, interteam cooperation is expected to promote team learning goal orientation and reduce team performance-prove and performance-avoid goal orientations.

In contrast, interteam competition is likely to foster team performance-prove and performance-avoid goal orientations and weaken team learning goal orientation. Interteam competition research demonstrated that interteam competition renders team members highly performance-oriented [40]. On one hand, as proposed by social identity theory, interteam competition strengthens team members’ identification with their team [37,41,42]. A high level of team identity motivates team members to strive for a positive team image. To boost their team image, team members seek to demonstrate the team’s competencies and excel other teams, which results in a collective performance-prove goal orientation.

On the other hand, interteam competition is also expected to enhance team performance-avoid goal orientation. This is grounded in the reasoning that a high level of conflict is associated with performance-avoid goal orientation [32]. When interteam competition results in conflicts between teams, team members tend to be afraid of failures, negative consequences, and threats to the reputation of their competence [13,43,44], which shapes a team-level performance-avoid goal orientation [24]. Moreover, a climate of negative emotions, triggered by interteam conflicts, can produce a pessimistic view of resource availability and may prompt team members to avoid outcomes that will aggravate negative emotions [45]. As such, conflicts and negative emotions resulting from interteam competition can cause team members to avoid negative evaluations, which strengthens a performance-avoid goal orientation within the team. 

On the contrary, interteam competition is presumed to reduce a learning goal orientation in the team. This is because competition between teams restricts the exchange and coordination of resources as well as communication among teams [8,41]. Such limited sharing of information and resources prevent teams from learning from each other [9,41], thereby stifling team learning goal orientation. Taken together, we put forth the following relationships:

**Hypothesis** **1.** *Interteam cooperation is positively related to (a) team learning goal orientation and negatively related to (b) team performance-prove goal orientation and (c) team performance-avoid goal orientation.*

**Hypothesis** **2.** *Interteam competition is negatively related to (a) team learning goal orientation and positively related to (b) team performance-prove goal orientation and (c) team performance-avoid goal orientation.*

### 3.2. Cross-Level Relationships between Team Goal Orientation and Boundary Activities

Drawing on the proposition that goal orientation shaped at the team level affects individual members’ goal-directed behaviors [13], we anticipate that all three team goal orientations are related to the three forms of boundary activities to some degree. Yet, we posit that the three types of team goal orientation are differentially associated with the three forms of boundary activities. Alexander and van Knippenberg’s [14] theoretical study offers crucial insights into such differential effects of team goal orientations on boundary activities. Alexander and van Knippenberg [14] proposed that teams with a learning orientation perform more technical scouting activities than teams with a performance-prove goal orientation, whereas teams with a performance-prove goal orientation engage in more ambassadorial activities than teams with a learning goal orientation. They argue that, while a team learning orientation motivates team members’ feedback-seeking regardless of whether feedback is positive or negative, a team performance-prove orientation promotes impression management activities to receive positive feedback.

Consistent with Alexander and van Knippenberg’s [14] propositions, we predict that, while both team learning and performance-prove goal orientations have a positive relationship with boundary spanning, there will be a stronger association between team learning goal orientation and boundary spanning than between team performance-prove goal orientation and boundary spanning. Associated with a desire to pursue a thorough and accurate understanding of the team’s task, a team learning goal orientation facilitates team members’ systematic information search, exchange, and processing [46]. Thus, members of a team with a learning goal orientation actively seek out information and learn from others [47,48]. Furthermore, because other teams can serve as a frame of reference for testing and improving the team’s ideas and knowledge, information exchange and knowledge sharing across team boundaries are frequently observed in teams with a learning goal orientation [16]. Indeed, team learning and performance-prove goal orientations are positively associated with information exchange (e.g., Reference [16]). However, team performance-prove goal orientation should lead to less boundary spanning than team learning goal orientation. Although teams with a performance-prove goal orientation also seek external feedback, their information exchange with other teams is limited because they consider feedback-seeking as a means for impression management and look for positive feedback [14]. On the other hand, due to fear of unfavorable competence judgments, teams with a performance-avoid goal orientation seldom seek feedback from the outside [14], which presumably leads to a negative association between team performance-avoid goal orientation and boundary spanning. Therefore, is the following is hypothesized:

**Hypothesis** **3.** *(a) Team learning goal orientation and (b) team performance-prove goal orientation are positively related to team members’ boundary spanning, whereas (c) team performance-avoid goal orientation is negatively related to team members’ boundary spanning. (d) Team learning goal orientation is more strongly related to team members’ boundary spanning than team performance-prove goal orientation.*

We propose that boundary reinforcement occurs most often in teams with a performance-prove goal orientation. This is because team performance-prove goal orientation is associated with strong team identity. When a performance-prove goal orientation is prevalent in a team, its members are heavily concerned about proving the team’s competencies and performance [49,50]. As team members constantly compare their performance with that of other teams [51], team boundaries become salient to them, which results in boundary-tightening rather than boundary-loosening behaviors. Salient team identity and boundaries can promote team performance by bonding team members toward goal achievement and focusing team members on the team’s task [3]. Given that setting boundaries and increasing member awareness of boundaries are beneficial to team performance [3], members of a team with a performance-prove goal orientation are likely to engage in boundary reinforcement behavior to enhance and demonstrate the team’s performance. Furthermore, as performance-oriented teams strive to outperform their competitors, they reinforce team boundaries by reducing resource leakage and enhancing cohesion and teamwork to achieve their goals [3]. Therefore, we expect a strong, positive relationship between team performance-prove goal orientation and boundary reinforcement. 

There is some evidence for the positive link between team learning and boundary reinforcement [3,52]. Given that salient boundaries strengthen team mission and commitment necessary for team learning activities [3], teams with a learning orientation tend to perform some degree of boundary reinforcement. However, boundary-tightening behaviors may limit opportunities for learning and growth. Because stressing too much team identity and cohesion may result in rigidity in team learning activities and inhibit information sharing with other teams, teams with a learning goal orientation are expected to engage in less boundary reinforcement than teams with a performance-prove goal orientation. As a result, we propose a stronger link between team performance-prove goal orientation and boundary reinforcement than between team learning goal orientation and boundary reinforcement.

In contrast, there should be a negative relationship between team performance-avoid goal orientation and boundary reinforcement. Teams with a performance-avoid goal orientation tend to avoid situations in which they need to demonstrate their competencies and compare their performance with other teams. In such teams, reinforcing team boundaries may render team members often exposed to comparison with other teams, which in turn leads to less boundary reinforcement. Taken together, we put forth the following hypothesis:

**Hypothesis** **4.** *(a) Team learning goal orientation and (b) team performance-prove goal orientation are positively related to team members’ boundary reinforcement, whereas (c) team performance-avoid goal orientation is negatively related to team members’ boundary reinforcement. (d) Team performance-prove goal orientation is more strongly related to team members’ boundary reinforcement than team learning goal orientation.*

Lastly, we postulate that there will be opposite relationships between team learning and performance-prove goal orientations and boundary buffering and between team performance-avoid goal orientation and boundary buffering. Performance-avoid goal orientation manifests as a tendency to avoid unfavorable evaluation [14]. Because comparisons with other teams pose the potential risk of receiving negative evaluations, teams with a performance-avoid goal orientation avoid communicating and exchanging information with other teams [15]. Furthermore, interactions with other teams are regarded as interferences and disturbances. Such teams tend to protect themselves by closing themselves off from exposure to the environment, and preventing members from interacting with other teams and releasing information to other teams [3]. However, insulation from the external environment is not desired by teams with learning and performance-prove goal orientations. Since information exchange and knowledge sharing with other teams are critical to team learning [16], it is unlikely that members of learning-oriented teams engage in boundary buffering. Likewise, the lack of interaction with the external environment hinders teams with a performance-prove goal orientation from receiving feedback on their performance and deprives them of opportunities to demonstrate their competencies outside the team. Therefore, teams with a performance-prove goal orientation are unlikely to engage in boundary buffering. This argumentation leads to the following hypothesis:

**Hypothesis** **5.** *(a) Team learning goal orientation and (b) team performance-prove goal orientation are negatively related to team members’ boundary buffering, whereas (c) team performance-avoid goal orientation is positively strongly related to team members’ boundary buffering.*

### 3.3. Cross-Level Mediation of Team Goal Orientation

Integrating the relationships proposed in the previous sections, we postulate that the three types of team goal orientation serve as crucial linking mechanisms between interteam cooperation and competition, and boundary activities. Drawing on Dragoni’s [13] cross-level model of state goal orientation, we propose that different team climates are posited to shape their relevant types of team goal orientation, which in turn affect individuals’ boundary activities. Although it is conceivable that the three types of team goal orientation are related to all three forms of boundary activities, based on the aforementioned hypotheses, a specific form of team goal orientation should have a stronger relationship with a particular boundary activity than the others. Thus, we predict that interteam cooperation facilitates team members’ boundary spanning by forming a team learning orientation. A cooperative interteam climate elicits a learning orientation that promotes the exchange of information, knowledge, and resources across team boundaries. In contrast, a competitive interteam climate is anticipated to be positively associated with boundary reinforcement and buffering through disparate mechanisms. On one hand, a competitive interteam climate strengthens a team’s orientation toward demonstrating its competencies. This performance-prove orientation propels team members to reinforce team boundaries and identities to attain favorable performance evaluations. On the other hand, competitive interteam relations can shape a collective orientation to avoid negative evaluations, which causes team members to protect their team from exposure to external disturbances by controlling the exchange of information and resources. Therefore, we put forth the following mediation hypotheses:

**Hypothesis** **6.** *Team learning goal orientation mediates the relationship between interteam cooperation and team members’ boundary spanning.*

**Hypothesis** **7.** *Team performance-prove goal orientation mediates the relationship between interteam competition and team members’ boundary reinforcement.*

**Hypothesis** **8.** *Team performance-avoid goal orientation mediates the relationship between interteam competition and team members’ boundary buffering.*

## 4. Study 1: Methods

### 4.1. Sample and Data Collection Procedure

We contacted 30 South Korean companies and invited them to participate in the research. To enhance the generalizability of study findings, we selected target companies through stratified sampling based on firm size, location, and industry. Of the 30 companies contacted, 24 companies agreed to participate in the research. The participating companies varied in size and industry (e.g., service, manufacturing, and banking/finance), and possessed a team-based structure where teams were the formal unit of goal setting and monitoring, resource allocation, and performance evaluation. as well as having one formal leader. The companies’ human resource personnel was solicited to select formal, permanent teams that displayed boundary work and to distribute surveys to the members of those teams. We excluded temporary or project teams because team climates and emergent states are unlikely to emerge in teams that exist for a short period of time.

The final sample consisted of 249 members in 45 teams. Average team size was 5.53 members, with team size ranging between two and 21 members. Participated teams varied in functions: managerial support (22%), strategies/planning (11%), sales/marketing (20%), finance/accounting (11%), and research and development (R&D) (22%). Of the respondents, 43% were female, and the average organizational and team tenure were 6.2 (SD = 6.7) and 3.4 (SD = 4.6) years, respectively. The respondents held different organizational positions: rank-and-file employees (50%), first-level supervisors (21%), managers (17%), and senior managers (11%). Their job functions included administration/sales (70%), production/engineering (14%), and research and development (R&D) (15%).

### 4.2. Measure

We measured all study variables on multi-item Likert scales (1 = *strongly disagree*, 5= *strongly agree*). Following Brislin’s [53] back-translation procedure, survey items originally written in English were translated into Korean and then back-translated into English to confirm the equivalence of the two versions. Because interteam cooperation and competition were operationalized as team members’ perceptions of cooperation and competition between their team and counterpart teams, their responses for these variables were aggregated to the team level, generating interteam cooperation and competition measures. In a similar vein, we aggregated team members’ responses for team goal orientation to the team level. The reliability of scales and aggregation statistics are reported below. To capture team-level properties, we used team-referent items for interteam cooperation and competition and team goal orientation.

**Interteam cooperation.** Interteam cooperation was assessed with the three-item scale of intergroup cooperation developed by Campion, Medsker, and Higgs [54] (α = 0.82, r_wg(j)_ = 0.85, ICC(1) = 0.24, ICC(2) = 0.64, F-statistics = 2.78, *p* < 0.001). Although ICC(2) values were below the cutoff of 0.70 in this study, scholars argue that high within-team agreement (*r*_wg(j)_ > 0.70) and between-team variability (significant F statistics) can justify data aggregation [55,56,57,58,59]. An example of the items was “my team cooperates with other teams to get the work done”.

**Interteam competition**. To measure interteam competition, we used four items (α = 0.76, r_wg(j)_ = 0.80, ICC(1) = 0.26, ICC(2) = 0.66, F-statistics = 2.95, *p* < 0.001) from Chen, Xie, and Chang’s [60] competitive orientation scale and modified them to interteam contexts. Chen et al.’s [60] scale consisted of seven items. However, we performed an exploratory factor analysis (EFA) and eliminated three items that exhibited factor loadings below 0.40 and cross-loaded on other factors. Sample items included “my team feels somewhat disappointed when other teams perform better than us” and “there is much competition between my team and other teams”. 

**Team learning goal orientation.** In agreement with prior research on team goal orientation (e.g., References [16,17,39]), we used four team-referent items (α = 0.88, r_wg(j)_ = 0.88, ICC(1) = 0.11, ICC(2) = 0.45, F-statistics = 1.80, *p* < 0.01) of VandeWalle’s [50] learning goal orientation scale. An example item was “my team often looks for opportunities to develop new skills and knowledge”.

**Team performance-prove goal orientation.** Team performance-prove goal orientation was assessed with four items (α = 0.87, r_wg(j)_ = 0.86, ICC(1) = 0.14, ICC(2) = 0.52, F-statistics = 2.17, *p* < 0.001) adapted from Vandewalle’s [50] performance-prove goal orientation scale [16,17]. A sample example was “my team is concerned with showing that it can perform better than other teams”. 

**Team performance-avoid goal orientation.** Parallel to the other two types of team goal orientation, team performance-avoid goal orientation was measured with three items (α = 0.84, r_wg(j)_ = 0.74, ICC(1) = 0.10, ICC(2) = 0.41, F-statistics = 1.71, *p* < 0.01) from Vandewalle’s [50] performance-avoid goal orientation scale [16,17]. One item was excluded from Vandewalle’s four-item scale due to cross-loading. An example of the items was “my team is concerned about taking on a task if its performance would reveal that it has low ability”.

**Boundary spanning.** To evaluate team members’ own boundary-spanning behavior, four items (α = 0.84) of Faraj and Yan’s [3] boundary-spanning scale were modified to self-referent items. An example of the items was “I solicit information and resources from elsewhere beyond my team”. 

**Boundary reinforcement.** Boundary reinforcement was assessed with four items (α = 0.77) adapted from Faraj and Yan’s [3] boundary-reinforcement scale. A sample item was “I share a common understanding of my team’s image or identity with my team members”. 

**Boundary buffering.** Boundary buffering was measured with three items (α = 0.72) derived from Faraj and Yan’s [3] boundary buffering scale. We excluded one item from Faraj and Yan’s [3] boundary buffering scale due to cross-loading. An example of the items was “I prevent outsiders from overloading my team with either too much information or too many requests”. 

**Control variables.** Given the multilevel structure of our data, we controlled for variables at different levels. At the individual level, team members’ characteristics that could affect their boundary activities (i.e., organizational tenure, job level, and job function) were controlled [4]. At the team level, team size (i.e., the number of members on the team) and team competency (four items, α = 0.82 [61]) were included as team-level controls in all subsequent analyses based on the boundary work and team literature (e.g., References [4,12,62,63,64]). At the organizational level, organizational size and industry were controlled. 

### 4.3. Analytic Strategies

As an analytic tool for testing multilevel relationships, we used hierarchical linear modeling (HLM) [65]. To test our hypothesis, we performed three-level HLM: individual (Level 1), team (Level 2), and organizational (Level 3) levels. To assess the proposed cross-level mediation hypotheses, we utilized Monte Carlo simulation procedure with a program written in R [66], which estimates confidence intervals more reliably than conventional bootstrapping methods of re-sampling [66,67]. 

## 5. Study 1: Results

Because all measures were obtained from the same respondents, we conducted a confirmatory factor analysis (CFA) on the items of eight variables using AMOS 22. As reported in Table 1, the hypothesized eight-factor model exhibited a good fit to the data (χ^2^ = 768.87, *df* = 406, comparative fit index (CFI) = 0.90, Tucker–Lewis index (TLI) = 0.88, root-mean-square error of approximation (RMSEA) = 0.06). Furthermore, the eight-factor model yielded a significantly better fit than the alternative models: Δχ^2^ (*df* = 7) = 114.61, *p* < 0.001 for the seven-factor model; Δχ^2^ (*df* = 13) = 436.26, *p* < 0.001 for the six-factor model; Δχ^2^ (*df* = 22) = 1014.44, *p* < 0.001 for the four-factor model; Δχ^2^ (*df* = 25) = 1254.76, *p* < 0.001 for the three-factor model; Δχ^2^ (*df* = 27) = 1435.81, *p* < 0.001 for the two-factor model; Δχ^2^ (*df* = 28) = 1684.42, *p* < 0.001 for the one-factor model. Thus, the measures used in Study 1 demonstrated sufficient discriminant validity.

Means, standard deviations, and intercorrelations are shown in Table 2. Hypotheses 1a, 1b, and 1c predicted a positive relationship between interteam cooperation and team learning goal orientation and a negative relationship between interteam cooperation and team performance-prove and performance-avoid goal orientations. Conversely, Hypotheses 2a, 2b, and 2c proposed a negative relationship between interteam competition and team learning goal orientation and a positive relationship between interteam competition and team performance-prove and performance-avoid goal orientations. These hypotheses were tested by regressing each team goal orientation on the team-level predictors and controls (Level 1) and organizational-level controls (Level 2). The results of this two-level HLM analysis are presented in Table 3. While interteam cooperation was positively associated with team learning goal orientation (γ = 0.35, *p* < 0.001), it had no relationship with team performance-prove (γ = 0.16, *p* = not significant (n.s.)) and performance-avoid goal orientations (γ = −0.09, *p* = n.s.). On the other hand, interteam competition was positively related to team performance-prove (γ = 0.71, *p* < 0.001) and performance-avoid goal orientations (γ = 0.45, *p* < 0.01), but exhibited no relationship with team learning goal orientation (γ = 0.13, *p* = n.s.). These findings provide support for Hypotheses 1a and 2b. 

Hypotheses 3a, 3b, and 3c postulated a positive relationship between team learning and performance-prove goal orientations and boundary spanning and a negative relationship between team performance-avoid goal orientation and boundary spanning. Hypothesis 3d further predicted a stronger relationship between team learning goal orientation and boundary spanning than between team performance-prove goal orientation and boundary spanning. The results of three-level HLM are depicted in Table 4. As demonstrated in Model 2 of Table 4, team learning goal (γ = 0.60, *p* < 0.01) and performance-prove goal orientations (γ = 0.47, *p* < 0.05) were positively associated with boundary spanning, whereas team performance-avoid goal orientation had a marginally negative relationship with boundary spanning (γ = −0.23, *p* < 0.10), which supports Hypotheses 3a, 3b, and 3c. In support of Hypothesis 3d, team learning goal orientation (γ = 0.60, *p* < 0.01) had a stronger relationship with boundary spanning than team performance-prove goal orientation (γ = 0.47, *p* < 0.05). 

Hypotheses 4a, 4b, and 4c proposed a positive relationship between team learning and performance-prove goal orientations and boundary reinforcement and a negative relationship between team performance-avoid goal orientation and boundary reinforcement. Hypothesis 4d further predicted a stronger relationship between team performance-prove goal orientation and boundary reinforcement than between team learning goal orientation and boundary reinforcement. As shown in Model 4 of Table 4, while team learning (γ = 0.56, *p* < 0.05) and performance-prove goal orientations (γ = 0.61, *p* < 0.01) were positively associated with boundary reinforcement, we detected no significant link between team performance-avoid goal orientation and boundary reinforcement (γ = −0.05, *p* = n.s.). Therefore, Hypotheses 4a and 4b were supported, whereas Hypothesis 4c received no support. In support of Hypothesis 4d, we found a stronger relationship between team performance-prove goal orientation and boundary reinforcement (γ = 0.61, *p* < 0.01) than between team learning goal orientation and boundary reinforcement (γ = 0.56, *p* < 0.05).

Hypotheses 5a, 5b, and 5c postulated a negative relationship between team learning and performance-prove goal orientations and boundary buffering and a positive relationship between team performance-avoid goal orientation and boundary buffering. As illustrated in Model 6 of Table 4, of the three goal orientations, only team learning-goal orientation had a marginally significant relationship with boundary buffering (γ = 0.60, *p* < 0.10), failing to support Hypotheses 5a, 5b, and 5c.

Hypotheses 6, 7, and 8 predicted the mediating effects of the three team goal orientations on the relationship between interteam cooperation and competition and the three forms of boundary activities. The results of the Monte Carlo simulation procedure (re-sampling = 20,000) are reported in Table 5. Interteam cooperation had a significant indirect effect on boundary spanning (estimate = 0.14, 95% confidence interval (CI) = 0.05, 0.26) and boundary reinforcement (estimate = 0.14, 95% CI = 0.02, 0.29) through team learning goal orientation. Interteam competition exerted a significant indirect effect on boundary spanning (estimate = 0.25, 95% CI = 0.05, 0.54) and boundary reinforcement (estimate = 0.35, 95% CI = 0.06, 0.46) through team performance-prove goal orientation. However, team performance-avoid goal orientation failed to mediate between interteam competition and boundary buffering (estimate = −0.07, 95% CI = −0.21, 0.02). These findings lend support to Hypotheses 6 and 7, but not to Hypothesis 8. 

## 6. Study 2: Method

### 6.1. Participants and Procedure

Because Study 1 relied on cross-sectional data, we could not ascertain the causality among the variables. In addition, although we controlled for important team characteristics such as team competency in Study 1, we needed to test our hypotheses by controlling for other team- and individual-level variables that might have a potential confounding effect on individual boundary activities. Thus, we conducted a two-wave longitudinal study to validate the proposed link between the three team goal orientations and the three forms of individual boundary activities. Study 2 was targeted at 188 undergraduate students at a large South Korean university who were enrolled in five introductory management courses (i.e., introduction to management, organizational behavior). The five courses were taught by the same instructor for two consecutive semesters. The instructor was blind to the study objectives and hypotheses, and the students who participated in the study received extra credit. As a course requirement, the students took part in an eight-week team project whose purpose was to generate solutions to real organizational problems by applying management principles. The results of their analysis were graded by the instructor and constituted 30% of the course grade. At the beginning of the project, the students were randomly assigned into a three-person team. Each week, teams held a 20-min in-class meeting to carry out their project. In the third week of the project (time 1: T1), we collected data on the students’ demographic information, own goal orientation, team goal orientation, and collective efficacy. In the eighth week (time 2: T2), when the teams submitted the final output of their project, we administered a T2 survey to assess team members’ boundary activities. Sixty-six percent of the students were male and 60% of the students were freshmen. Their average age was 19.7 years (SD = 1.7).

### 6.2. Measures

Similar to Study 1, a back-translation procedure [53] was used and all variables were measured on a five-point Likert scale (1 = strongly disagree, 5 = strongly agree). 

**Team goal orientation (T1).** As a measure of team goal orientation, we adapted Vandewalle’s [50] goal orientation scale as team-referent items. Of the original 12 items assessing learning, performance-prove, and performance-avoid goal orientations, we eliminated three items due to cross-loadings, which resulted in three-item scale of team learning goal orientation (α = 0.78, r_wg(j)_ = 0.77, ICC(1) = 0.21, ICC(2) = 0.44, F = 1.80, *p* < 0.01), three-item scale of team performance-prove goal orientation (α = 0.67, r_wg(j)_ = 0.83, ICC(1) = 0.19, ICC(2) = 0.42, F = 1.71, *p* < 0.01), and three-item scale of team performance-avoid goal orientation (α = 0.65, r_wg(j)_ = 0.73, ICC(1) = 0.18, ICC(2) = 0.40, F = 1.68, *p* < 0.01).

**Boundary activities (T2).** To assess boundary activities, we modified Faraj and Yan’s [3] boundary activity scale to self-referent items. Of the original 12 items assessing boundary spanning, reinforcement, and buffering, two items were excluded because of cross-loadings. As a result, our boundary activities scale consisted of three items of boundary spanning (α = 0.77), four items of boundary reinforcement (α = 0.77), and three items of boundary buffering (α = 0.60). 

**Control variables.** We controlled for variables at different levels. At the individual level, we controlled for team members’ age and gender, as well as their own learning, performance-prove, and performance-avoid goal orientations. At the team level, collective efficacy (four items, α = 0.75 [68]) were included as a team-level control due to its potential effect on boundary activities [4,12]. Additionally, because participants were enrolled in two courses, a course dummy (1 = organizational behavior, 0 = others) was created as a course-level control. 

## 7. Study 2: Results

To assess the discriminant validity among the study variables, we performed a CFA on the items of six variables. The hypothesized six-factor structure exhibited a good fit to the data in an absolute sense (χ^2^ (*df* = 137) = 182.04, CFI = 0.94, TLI = 0.94, RMSEA = 0.04) and fitted the data significantly better than the three-factor model (combining team learning goal orientation and boundary spanning into a single factor, team performance-prove goal orientation and boundary reinforcement into a single factor, and team performance-avoid goal orientation and boundary buffering into a single factor; χ^2^ (*df* = 149) = 549.94, CFI = 0.51, TLI = 0.38, RMSEA = 0.11) and the two-factor model (combining three types of team goal orientation into a single factor and three forms of boundary activities into a single factor; χ^2^ (*df* = 151) = 490.16, CFI = 0.59, TLI = 0.48, RMSEA = 0.10), which confirmed the discriminant validity of the focal measures.

**Figure 2:**
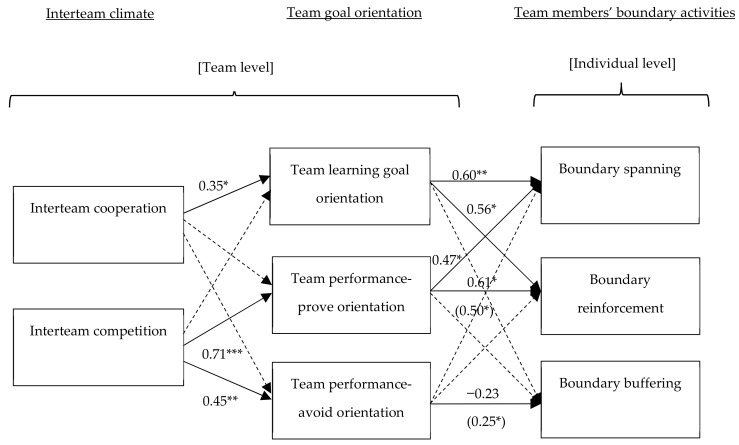
Summary of results. Notes. Standardized coefficients are reported. Numbers in parentheses represent significant coefficients in Study 2. * *p* < 0.05, ** *p* < 0.01, *** *p* < 0.001.

Means, standard deviations, and intercorrelations are presented in Table 6. Each form of boundary activities was regressed on individual-, team-, and course-level predictors. The results of this three-level HLM are reported in Table 7. Contrary to our prediction, T1 team learning goal orientation was not related to T2 boundary spanning (γ = −0.16, *p* = n.s.) and boundary reinforcement (γ = 0.18, *p* = n.s.). As proposed, T1 team performance-prove goal orientation was positively associated with T2 boundary reinforcement (γ = 0.50, *p* < 0.05). T1 team performance-prove goal orientation (γ = 0.50, *p* < 0.05) had a stronger relationship with T2 boundary reinforcement than T1 team learning goal orientation (γ = 0.18, *p* = n.s.). These findings provide support for Hypotheses 4b and 4d. In addition, we found that T1 team performance-avoid goal orientation was positively related to T2 boundary buffering (γ = 0.25, *p* < 0.05), which supports Hypothesis 5c. The results of Studies 1 and 2 are summarized in Figure 2.

## 8. Discussion

The purpose of this study was to propose and test the cross-level mediation of team goal orientation on the relationships between interteam cooperation and competition and different forms of boundary activities. In Study 1, we detected positive associations between interteam cooperation and team learning goal orientation and between interteam competition and team performance-prove and performance-avoid goal orientations. We found that both team learning and performance-prove goal orientations were positively related to boundary spanning and reinforcement. As predicted, team learning goal orientation had a stronger relationship with boundary spanning than team performance-prove goal orientation, whereas team performance-prove goal orientation was more strongly related to boundary reinforcement than team learning goal orientation. Team learning goal orientation mediated the relationship between interteam cooperation and boundary spanning and reinforcement, whereas team performance-prove goal orientation mediated the link between interteam competition and boundary spanning and reinforcement. The results of Study 2 indicated the positive lagged effects of team performance-prove goal orientation on boundary reinforcement and of team performance-avoid goal orientation on boundary buffering. These findings offer important implications for the boundary work literature.

### 8.1. Theoretical Implications

While there is a growing body of research on the antecedents of boundary activities, the mediating processes by which interteam cooperation and competition are linked to boundary activities remain unclear. Given that interteam cooperation and competition are common in team-based organizations, exploring the relationship between interteam cooperation and competition and boundary activities, as well as their intermediary processes, has both theoretical and practical value. Our results demonstrate that interteam cooperation contributes to boundary spanning and reinforcement by shaping team learning goal orientation. In contrast, interteam competition leads to boundary spanning and reinforcement by eliciting team performance-prove goal orientation. These findings corroborate prior findings that indicate a positive link between interteam interdependence or cooperation and boundary spanning (e.g., References [5,8]), as well as a positive relationship between team learning goal orientation and boundary spanning (e.g., Reference [14]). In addition, these findings endorse Dragoni’s [13] cross-level model of state goal orientation by revealing team goal orientation as an intermediary mechanism connecting team climate and individual behavior. 

Our findings suggest that although both interteam cooperation and competition were positively associated with boundary spanning and reinforcement, this is realized though the distinct mediating processes of different team goal orientations. As depicted in Table 4, both interteam cooperation and competition had a direct, positive relationship with the two forms of boundary activities. However, when the mediators were entered in the HLM equation, the relationships between interteam cooperation and competition and the two forms of boundary activities became weaker or insignificant, suggesting partial or full mediation. More precisely, interteam cooperation increases team members’ boundary spanning by shaping a team learning goal orientation, whereas interteam competition enhances boundary reinforcement by evoking a collective orientation to demonstrate the team’s competencies. Although not hypothesized, there was a significant mediating effect of team learning goal orientation on the relationship between interteam cooperation and boundary reinforcement, which implies two distinct routes leading to boundary reinforcement. Likewise, team performance-prove goal orientation exerted a significant mediating effect on the relationship between interteam competition and boundary spanning. These findings are consistent with prior findings suggesting that both team learning and performance-prove goal orientations are beneficial to teams’ information exchange and learning (e.g., Reference [16]). Expanding this line of research, our findings further reveal that even though both team learning and performance-goal orientation are positively associated with boundary spanning and reinforcement, the two team goal orientations result from different interteam dynamics. As such, by revealing different pathways through which interteam cooperation and competition induce boundary spanning and reinforcement, this study adds fine-grained knowledge to the boundary work literature. 

Contrary to our prediction, we detected a positive link between interteam cooperation and boundary buffering, as well as a marginally significant association between team learning goal orientation and boundary buffering. This was quite surprising because we anticipated that team learning goal orientation would decrease boundary buffering. One possible explanation for this unexpected finding is that, when teams collaborate with one another on interdependent tasks, team members may encounter conflicting priorities and demands from multiple teams and, therefore, may protect themselves from external pressure or disturbances [3]. In particular, when team members focus on learning, they may need to insulate themselves from external interferences in order to concentrate on learning activities such as idea incubation and reflection. Therefore, teams with a learning orientation may engage in boundary buffering. However, because the marginally significant relationship between team learning goal orientation and boundary buffering was not replicated in Study 2, the potential impact of team learning goal orientation on boundary buffering warrants future empirical investigations. 

Our research offers important insights for the goal orientation literature by treating team goal orientation as a team-level state that promotes team members’ boundary activities and delineating the differential effects of different team goal orientations on boundary activities. Our findings clearly demonstrate that, even when controlling for important team characteristics (i.e., team competency and collective efficacy) and individuals’ own goal orientation, team goal orientation significantly predicts its relevant form of boundary activities. While prior research generally focused on goal interdependence among teams as a precondition for boundary activities (e.g., References [5,8]), the role of shared goals within a team for predicting boundary activities was rarely explored. In addition to the structure in which team goals are related to one another in interteam dynamics, goal orientations collectively pursued by team members can affect their boundary activities. This implies that internal team processes, or emergent states within the team can play an important role on boundary activities; the goal orientation adopted by the team as a whole can influence the type of boundary activities that individual team members carry out. In particular, in Study 2, team goal orientation predicted individuals’ boundary activities more strongly than their own goal orientation did. Thus, by shifting the locus of goals critical to boundary activities from interteam relations to within-team contexts, this study significantly expands the boundary work and goal orientation literature. 

In addition, our findings demonstrate that there is a type of team goal orientation that is more strongly related to a specific form of boundary activities. In Study 1, team learning goal orientation was most strongly related to boundary spanning. In Studies 1 and 2, team performance-prove goal orientation had the strongest effect on boundary reinforcement. In Study 2, team performance-avoid goal orientation had the strongest relationship with boundary buffering. These findings endorse Alexander and van Knippenberg’s [14] proposition that team learning and performance-prove goal orientations are differentially associated with different forms of boundary activities. The differential relationships between the three team goal orientations and boundary activities were partially validated in Study 2. In Study 2, we observed the significant lagged effects of team performance-prove goal orientation (T1) on boundary reinforcement (T2) and of team performance-avoid goal orientation (T1) on boundary buffering (T2), which suggest the causal relationship between team goal orientation and boundary activities. However, the positive relationship between team performance-avoid goal orientation and boundary buffering. as well as the mediating effect of team performance-avoid goal orientation were not supported in Study 1. These inconsistent findings for team performance-avoid goal orientation might be due to the different natures between intact work teams and student project teams. Unlike student teams, work teams in organizations are highly performance-driven, with their performance being a key criterion for important human resource decisions. Under such a circumstance, team members tend to seek opportunities to demonstrate their performance and abilities rather than avoid opportunities to demonstrate their competencies. Thus, performance-avoid goal orientation is generally deemed as insignificant by teams in results-oriented environments [17], which might have attenuated the role of team performance-avoid goal orientation in Study 1. 

### 8.2. Practical Implications

The present research provides several practical implications for managers and team leaders. Interteam cooperation and competition are prevalent in today’s organizations whose basic work units are teams. The present findings have meaningful implications for managing goals and boundary activities by uncovering the linkages between interteam cooperation and competition, team goal orientations, and individual boundary activities. Given that both interteam cooperation and competition are contextual factors promoting boundary spanning and reinforcement, increasing interteam cooperation and competition can help teams engage in more boundary spanning and reinforcement. Scholars recommend interdependent goals as a means to facilitate cooperation among teams [5,69]. Assigning teams interdependent goals can build a cooperative climate by stimulating communication and interactions among teams. Designing and implementing reward systems contingent on interdependent goals can also foster interteam collaboration. Furthermore, formulating and emphasizing collective organizational objectives and providing teams with feedback related to those objectives serve to elevate the level of goal interdependence among teams [8]. However, given that interteam competition is a double-edged sword that has a positive ramification on group cohesion and performance and a negative impact on member well-being [70,71], managers and team leaders should exercise caution when instigating interteam competition. 

Based on our findings, managers and team leaders are advised to take a balanced approach to factors affecting boundary activities. Our findings indicate that while both interteam cooperation and competition are positively related to boundary spanning and reinforcement, they affect these activities through disparate mechanisms. While team learning goal orientation promotes boundary spanning and reinforcement in a cooperative interteam climate, team performance-prove goal orientation plays a more influential role in boundary spanning and reinforcement in a competitive interteam climate, which implies that different goal strategies are called for depending on whether the interteam climate is cooperative or competitive. When there is fierce interteam competition, team leaders may need to foster a performance-prove goal orientation in their team to increase boundary spanning and reinforcement. Conversely, in high interteam cooperation situations, shaping a team learning goal orientation can be a better strategy for promoting boundary spanning and reinforcement. 

### 8.3. Limitations and Directions for Future Research

Despite its theoretical and practical implications, this study is not without limitations. Firstly, because all variables were measured simultaneously in Study 1, causal conclusions about the proposed mediating effects should be made with caution. Although we validated the causal relationship between team goal orientation and boundary activities by using two-wave data in Study 2, the causal relationship between interteam cooperation and competition and team goal orientation was not established in our studies. Thus, it is plausible that a high level of team learning goal orientation leads to cooperative interteam climate. Likewise, performance-prove or performance-avoid goal orientation that resides in a team may create a competitive interterm climate. Therefore, the causal direction proposed in our research needs to be validated in future research that employs more rigorous methodology such as longitudinal, panel, or experimental designs [72]. 

Another methodological limitation of our study is that all variables were measured by the same respondents. Because we used aggregated responses for interteam climates and team goal orientations in Study 1, the correlations between interteam cooperation and team learning goal orientation and between interteam competition and team performance-prove and performance-avoid goal orientations might have been inflated due to common method biases [73]. To resolve this issue, it is desirable to obtain the measures of variables from different sources [74]. For instance, the target employee’s peer or immediate supervisor can provide an objective assessment of his or her boundary activities. 

Although social identity theory was presented as an explanatory framework for the relationship between interteam cooperation and competition and team goal orientation, identity-related variables were neither measured nor analyzed in this study. To better explain the role of social identity in boundary activities, future work could be directed at testing the linkage among interteam relations, social identity, team goal orientation, and boundary activities. Furthermore, while we explored the antecedents and mediating processes affecting boundary activities and their differential relationships, we did not investigate which form of boundary activity is more or less beneficial to team effectiveness. While there is some evidence for the positive effect of boundary spanning, reinforcement, and buffering on team performance (e.g., Reference [3]), more knowledge should be accumulated with respect to the differential relationship between boundary activities and more diverse team outcomes. Thus, we recommend that future research explore the consequences of different forms of boundary activities on diverse team outcomes. 

Finally, failure to consider boundary conditions is another study limitation. There was a research call for the variables that may have a moderating effect on boundary activities [2,3]. In a similar vein, Drach-Zahavy and Somech [8] proposed team heterogeneity, team power, organizational culture, and environmental conditions as boundary conditions affecting the relationship between interteam relations and boundary activities. Furthermore, building on the proposition that leadership has a critical influence on team goal orientation [13] and boundary activities [2,75], it would be interesting for future research to explore the moderating role that leadership plays in the relationship between team goal orientation and boundary activities. 

## 9. Conclusions

In conclusion, our study offers novel insights for boundary activities research by being the first to reveal team learning and performance-prove goal orientations as crucial mediating mechanisms by which interteam cooperation and competition affect team members’ boundary spanning and reinforcement. While our study focused on boundary activities directed toward other teams, follow-up research may need to assess the role of goal orientation in predicting a broader scope of boundary activities that occur at different levels of organizations. Moreover, for a more rigorous test of Dragoni’s [13] model, it is necessary to elucidate how team and individual goal orientations interactively affect boundary activities taking place at different organizational levels. Future work investigating such issues can disentangle the complicated multilevel dynamics involving boundary activities at different levels.

## Figures and Tables

**Table 1:** Confirmatory factor analyses and chi-square difference tests (Study 1).

Models	χ^2^	*df*	Δχ^2^	CFI	TLI	RMSEA
Model 0. Eight-factor model	768.87	406	-	0.90	0.88	0.06
Model 1. Seven-factor model (combines team performance-prove goal orientation and interteam competition into a single factor)	883.48	413	114.61 ***	0.87	0.85	0.06
Model 2. Six-factor model (combines team performance-prove and performance-avoid goal orientation into a single factor, and boundary reinforcement and buffering into a single factor)	1205.13	419	436.26 ***	0.78	0.76	0.08
Model 3. Four-factor model (combines three types of team goal orientation into a single factor, and three types of boundary activities into a single factor)	1783.31	428	1014.44 ***	0.63	0.60	0.11
Model 4. Three-factor model (combines interteam cooperation and competition into a single factor, three types of team goal orientation into a single factor, and three types of boundary activities into a single factor)	2023.63	431	1254.76 ***	0.57	0.53	0.12
Model 5. Two-factor model (combines interteam cooperation and competition into a single factor, and three types of team goal orientation and three types of boundary activities into a single factor)	2204.68	433	1435.81 ***	0.52	0.48	0.12
Model 6. One-factor model	2453.29	434	1684.42 ***	0.45	0.41	0.13

**Notes.***N* = 249. CFI = comparative fit index, TLI = Tucker–Lewis index, RMSEA = root-mean-square error of approximation. *** *p* < 0.001.

**Table 2:** Descriptive statistics and intercorrelations (Study 1).

	Mean	SD	1	2	3	4	5	6
*Organizational level* ^a^								
1. Organizational size dummy	0.50	0.51	-					
2. Industry dummy 1	0.29	0.46	0.09	-				
3. Industry dummy 2	0.16	0.38	0.44 *	−0.28	-			
*Team level* ^b^								
1. Team size	5.53	3.65	-					
2. Team competency	3.62	0.36	0.18	-				
3. Interteam cooperation	3.88	0.48	0.05	0.17	-			
4. Interteam competition	2.95	0.53	−0.00	0.27	−0.05	-		
5. TLGO	3.83	0.43	0.07	0.29 *	0.50 ***	0.13	-	
6. TPPGO	3.55	0.49	0.12	0.27	0.10	0.77 ***	0.27	-
7. TPAGO	3.20	0.50	0.00	−0.23	−0.14	0.27	−0.12	0.21
*Individual level* ^c^								
1. Organizational tenure	6.17	6.71	-					
2. Job level	0.28	0.45	0.22 ***	-				
3. Job function dummy 1	0.14	0.34	0.35 ***	−0.04	-			
4. Job function dummy 2	0.15	0.36	−0.21 **	−0.07	−0.17 **	-		
5. Boundary spanning	3.62	0.70	−0.08	0.02	−0.00	0.03	-	
6. Boundary reinforcement	3.72	0.66	−0.14 *	0.04	−0.05	0.00	0.50 ***	-
7. Boundary buffering	3.39	0.72	−0.06	−0.04	−0.03	−0.01	0.47 ***	0.38 ***

**Notes.**^a^*N* = 24, ^b^ *N* = 45, ^c^ *N* = 249. Organizational size dummy: 1 = above 300 employees, 0 = below 300 employees. Industry dummy 1: manufacturing = 1, others = 0. Industry dummy 2: finance = 1, others = 0. Job function dummy 1: 1 = production/engineering, 0 = others. Job function dummy 2: 1 = research and development (R&D), 0 = others. TLGO = team learning goal orientation. TPPGO = team performance-prove goal orientation. TPAGO = team performance-avoid goal orientation. Job level: 1 = managers, 0 = non-managers. + *p* < 0.10, * *p* < 0.05, ** *p* < 0.01, *** *p* < 0.001.

**Table 3:** Two-level hierarchical linear modeling for team goal orientation (Study 1).

*Variables*	Team Learning Goal Orientation		Team Performance-Prove Goal Orientation		Team Performance-Avoid Goal Orientation	
	Model 1	Model 2	Model 3	Model 4	Model 5	Model 6
*Organizational level* ^a^						
Organizational size dummy	0.01	0.03	−0.24	−0.19	−0.19	−0.06
Industry dummy 1	−0.51 +	−0.45 +	0.01	0.10	−0.46	−0.44
Industry dummy 2	0.24	0.08	0.82	1.07 **	−0.62	−0.28
*Team level* ^b^						
Team size	−0.00	−0.00	0.01	0.00	−0.00	−0.00
Team competency	0.47 *	0.31 *	0.59 *	0.17	−0.40	−0.51 *
Interteam cooperation		0.35 ***		0.16		−0.09
Interteam competition		0.13		0.71 ***		0.45 **
Within-organization variance	0.08	0.13	0.19	0.12	0.22	0.15
Between-organization variance	0.10	0.04	0.06	0.00	0.01	0.06
Deviance	51.66	41.50	70.36	47.78	71.01	66.18

**Notes.**^a^*N* = 24, ^b^ *N* = 45. Organizational size dummy: 1 = above 300 employees, 0 = below 300 employees. Industry dummy 1: manufacturing = 1, others = 0. Industry dummy 2: finance = 1, others = 0. Job level: 1 = managers, 0 = non-managers. Job function dummy 1: 1 = production/engineering, 0 = others. Job function dummy 2: 1 = R&D, 0 = others. + *p* < 0.10, * *p* < 0.05, ** *p* < 0.01, *** *p* < 0.001.

**Table 4:** Three-level hierarchical linear modeling for boundary activities (Study 1).

*Variables*	Boundary Spanning		Boundary Reinforcement		Boundary Buffering	
	Model 1	Model 2	Model 3	Model 4	Model 5	Model 6
*Organizational level* ^a^						
Organization size dummy	−0.30 *	−0.19 +	−0.35 +	−0.14	0.00	0.07
Industry dummy 1	0.26	0.14	−0.13	−0.09	0.02	−0.07
Industry dummy 2	1.13 *	0.35	0.78	−0.12	1.21 +	0.81 +
*Team level* ^b^						
Team size	0.00	0.00	0.00	0.00	−0.00	−0.00
Team competency	−0.44	−0.73 *	0.03	−0.07	−0.96 ***	−1.14 ***
Interteam cooperation	0.58 **	0.27 *	0.50 **	0.28 +	0.54 **	0.30+
Interteam competition	0.27 +	0.00	0.28 **	−0.18	0.17	0.29
TLGO		0.60 **		0.56 *		0.60+
TPPGO		0.47 *		0.61 **		−0.11
TPAGO		−0.23 +		−0.05		−0.23
*Individual level* ^c^						
Organizational tenure	−0.00 +	−0.00	−0.00	−0.00	−0.00	−0.00
Job level	0.17	0.17	0.17	0.17	0.11	0.11
Job function dummy 1	0.70 **	0.70 **	0.69 **	0.69 **	0.76 **	0.76 **
Job function dummy 2	0.15	0.15	0.20	0.20	0.26	0.26
Within-team variance	0.42	0.41	0.34	0.33	0.44	0.44
Between-team variance	0.02	0.00	0.00	0.00	0.02	0.00
Between-organization variance	0.00	0.00	0.03	0.00	0.00	0.00
Model deviance	494.97	479.73	452.53	436.00	510.42	500.59

**Notes.**^a^*N* = 24, ^b^ *N* = 45, ^c^ *N* = 249. Organizational size dummy: 1 = above 300 employees, 0 = below 300 employees. Industry dummy 1: manufacturing = 1, others = 0. Industry dummy 2: finance = 1, others = 0. Job function dummy 1: 1 = production/engineering, 0 = others. Job function dummy 2: 1 = R&D, 0 = others. TLGO = team learning goal orientation. TPPGO = team performance-prove goal orientation. TPAGO = team performance-avoid goal orientation. Job level: 1 = managers, 0 = non-managers. + *p* < 0.10, * *p* < 0.05, ** *p* < 0.01, *** *p* < 0.001.

**Table 5:** Test of indirect effect (Study 1).

	Estimate	Low Level 95% CI	Upper Level 95% CI
Interteam cooperation → TLGO → Boundary spanning	0.14	0.05	0.26
Interteam cooperation → TLGO → Boundary reinforcement	0.14	0.02	0.29
Interteam competition → TPPGO → Boundary spanning	0.25	0.05	0.54
Interteam competition → TPPGO → Boundary reinforcement	0.35	0.06	0.46
Interteam competition → TPAGO → Boundary buffering	−0.07	-0.21	0.02

**Notes.** TLGO = team learning goal orientation. TPPGO = team performance-prove goal orientation. TPAGO = team performance-avoid goal orientation. CI = confidence interval.

**Table 6:** Descriptive statistics and intercorrelations (Study 2).

	Mean	S.D.	1	2	3	4	5	6	7
*Team level* ^a^									
1. Collective efficacy	3.46	0.45	-						
2. TLGO (T1)	3.94	0.44	0.17	-					
3. TPPGO (T1)	3.81	0.46	0.25 *	0.55 ***	-				
4. TPAGO (T1)	3.51	0.48	−0.04	0.27 *	0.33 **	-			
*Individual level* ^b^									
1. Age	19.74	1.68	-						
2. Gender	0.66	0.47	0.26 ***	-					
3. LGO	3.86	0.80	0.06	0.06	-				
4. PPGO	3.51	0.83	−0.08	0.05	0.02	-			
5. PAGO	3.29	0.85	−0.07	−0.03	0.04	0.00	-		
6. Boundary spanning (T2)	3.23	0.81	−0.09	−0.03	0.07	0.05	0.00	-	
7. Boundary reinforcement (T2)	3.98	0.64	0.15 *	0.03	0.14 *	−0.01	−0.15 *	0.06	-
8. Boundary buffering (T2)	3.51	0.60	−0.07	0.00	0.08	0.13 +	-0.06	0.23 **	0.23 **

**Notes.**^a^*N* = 66, ^b^ *N* = 188. Gender: 1 = male, 0 = female. TLGO = team learning goal orientation. TPPGO = team performance-prove goal orientation. TPAGO = team performance-avoid goal orientation. LGO = learning goal orientation. PPGO = performance-prove goal orientation. PAGO = performance-avoid goal orientation. T1 = time 1. T2 = time 2. + *p* < 0.10, * *p* < 0.05, ** *p* < 0.01, *** *p* < 0.001.

**Table 7:** Three-level hierarchical linear modeling for boundary activities (Study 2).

*Variables*	Boundary Spanning(T2)	Boundary Reinforcement(T2)	Boundary Buffering (T2)
	Model 1	Model 2	Model 3
*Course level* ^a^			
Course dummy	−0.19	0.37 +	−0.19
*Team level* ^b^			
Collective efficacy	0.12	0.31 +	0.26 +
TLGO (T1)	−0.16	0.18	−0.01
TPPGO (T2)	0.38	0.50 *	0.09
TPAGO (T3)	0.00	−0.06	0.25 *
*Individual level* ^c^			
Age	−0.04	−0.00	0.00
Gender	−0.12	0.12	0.10
LGO (T1)	0.05	0.03	0.06
PPGO (T1)	0.04	0.03	0.05
PAGO (T1)	−0.01	−0.07 *	−0.02
Within-team variance	0.62	0.24	0.32
Between-team variance	0.00	0.07	0.00
Between-organization variance	0.00	0.00	0.00
Model deviance	420.44	295.04	304.97

**Notes.**^a^*N* = 5, ^b^ *N* = 66, ^c^ *N* = 188. Course dummy: 1 = organizational behavior, 0 = others. Gender: 1 = male, 0 = female. TLGO = team learning goal orientation. TPPGO = team performance-prove goal orientation. TPAGO = team performance-avoid goal orientation. LGO = learning goal orientation. PPGO = performance-prove goal orientation. PAGO = performance-avoid goal orientation. T1 = time 1. T2 = time 2. + *p* < 0.10, * *p* < 0.05, ** *p* < 0.01, *** *p* < 0.001.

## References

[1] Ancona D.G., Caldwell D. (1990). Beyond boundary spanning: Managing external dependence in product development teams. J. High. Technol. Manag. Res..

[2] Choi J.N. (2002). External activities and team effectiveness: Review and theoretical development. Small Group Res..

[3] Faraj S., Yan A. (2009). Boundary work in knowledge teams. J. Appl. Psychol..

[4] Marrone J.A., Tesluk P.E., Carson J.B. (2007). A Multilevel Investigation of Antecedents and Consequences of Team Member Boundary-Spanning Behavior. Acad. Manag. J..

[5] Somech A., Khalaili A. (2014). Team boundary activity: Its mediating role in the relationships between structural conditions and team innovation. Group Organ. Manag..

[6] Dey C., Ganesh M.P. (2017). Team boundary activity: A review and directions for future research. Team Perform. Manag. Int. J..

[7] Gladstein D.L. (1984). Groups in Context: A Model of Task Group Effectiveness. Adm. Sci. Q..

[8] Drach-Zahavy A., Somech A. (2010). From an Intrateam to an Interteam Perspective of Effectiveness: The Role of Interdependence and Boundary Activities. Small Group Res..

[9] Joshi A., Pandey N., Han G. (2009). Bracketing team boundary spanning: An examination of task-based, team-level, and contextual antecedents. J. Organ. Behav..

[10] De Vries T.A., Walter F., Essens P.J.M.D., Van Der Vegt G.S. (2014). Antecedents of Individuals’ Interteam Coordination: Broad Functional Experiences as a Mixed Blessing. Acad. Manag. J..

[11] Maxwell-Smith M.A., Barnes K.L., Wright J.D., Thomson C., Mattos M.A., Dumas T.M. (2016). Competition and intergroup bias: Toward a new construal process framework distinguishing competitive perception from competitive motivations. Group Process. Intergroup Relat..

[12] Richter A.W., West M.A., Van Dick R., Dawson J.F. (2006). Boundary Spanners’ Identification, Intergroup Contact, and Effective Intergroup Relations. Acad. Manag. J..

[13] Dragoni L. (2005). Understanding the Emergence of State Goal Orientation in Organizational Work Groups: The Role of Leadership and Multilevel Climate Perceptions. J. Appl. Psychol..

[14] Alexander L., Van Knippenberg D. (2014). Teams in Pursuit of Radical Innovation: A Goal Orientation Perspective. Acad. Manag. Rev..

[15] Deshon R.P., Kozlowski S.W.J., Schmidt A.M., Milner K.R., Wiechmann D. (2004). A Multiple-Goal, Multilevel Model of Feedback Effects on the Regulation of Individual and Team Performance. J. Appl. Psychol..

[16] Gong Y., Kim T.-Y., Lee D.-R., Zhu J. (2013). A Multilevel Model of Team Goal Orientation, Information Exchange, and Creativity. Acad. Manag. J..

[17] Mehta A., Feild H., Armenakis A., Mehta N. (2009). Team Goal Orientation and Team Performance: The Mediating Role of Team Planning. J. Manag..

[18] Marrone J.A. (2010). Team boundary spanning: A multilevel review of past research and proposals for future research. J. Manag..

[19] Cross R.L., Yan A., Louis M.R. (2000). Boundary Activities in ‘Boundaryless’ Organizations: A Case Study of a Transformation to a Team-Based Structure. Hum. Relat..

[20] Drach-Zahavy A. (2011). Inter-organizational teams as boundary spanners: The role of team diversity, boundedness, and extra-team links. Eur. J. Work Organ. Psychol..

[21] Guinan P.J., Cooprider J.G., Faraj S. (1998). Enabling Software Development Team Performance During Requirements Definition: A Behavioral Versus Technical Approach. Inf. Syst. Res..

[22] Sawyer S., Guinan P.J., Cooprider J. (2010). Social interactions of information systems development teams: A performance perspective. Inf. Syst. J..

[23] Baer M., Leenders R.T.A.J., Oldham G.R., Vadera A.K. (2010). Win or Lose the Battle for Creativity: The Power and Perils of Intergroup Competition. Acad. Manag. J..

[24] Deutsch M. (1949). A Theory of Co-operation and Competition. Hum. Relat..

[25] Ashforth B.E. (1985). Climate Formation: Issues and Extensions. Acad. Manag. Rev..

[26] Liao H., Rupp D.E. (2005). The Impact of Justice Climate and Justice Orientation on Work Outcomes: A Cross-Level Multifoci Framework. J. Appl. Psychol..

[27] Mayer D.M., Kuenzi M., Greenbaum R., Bardes M., Salvador R. (2009). How low does ethical leadership flow? Test of a trickle-down model. Organ. Behav. Hum. Decis. Process..

[28] Naumann S.E., Bennett N. (2000). A Case for Procedural Justice Climate: Development and Test of a Multilevel Model. Acad. Manag. J..

[29] Salancik G.R., Pfeffer J. (1978). A Social Information Processing Approach to Job Attitudes and Task Design. Adm. Sci. Q..

[30] Beersma B., Hollenbeck J.R., Humphrey S.E., Moon H., Conlon D.E., Ilgen D.R. (2003). Cooperation, Competition, and Team Performance: Toward A Contingency Approach. Acad. Manag. J..

[31] Johnson D.W. (2003). Social Interdependence: Interrelationships Among Theory, Research, and Practice. Am. Psychol..

[32] Maltarich M.A., Greenwald J., Reilly G. (2016). Team-level goal orientation: An emergent state and its relationships with team inputs, process, and outcomes. Eur. J. Work. Organ. Psy..

[33] Chi N., Huang J. (2014). Mechanisms linking transformational leadership and team performance: The mediating roles of team goal orientation and group affective tone. Group Organ. Manag..

[34] Hoegl M., Weinkauf K., Gemuenden H.G. (2004). Interteam Coordination, Project Commitment, and Teamwork in Multiteam R&D Projects: A Longitudinal Study. Organ. Sci..

[35] Tajfel H., Tajfel H. (1978). The achievement of group differentiation. Differentiation between Social Groups: Studies in the Social Psychology of Intergroup Relations.

[36] Tajfel H., Turner J.C., Worchel S., Austin W.G. (1986). The social identity theory of intergroup behavior. Psychology of Intergroup Relations.

[37] Ashforth B.E., Mael F., Sonnenfeld J.A., Peiperl M.A. (1989). Social Identity Theory and the Organization. Acad. Manag. Rev..

[38] Bornstein G., Erev I. (1994). The enhancing effect of intergroup competition on group performance. Int. J. Confl. Manag..

[39] Kramer R.M., Brewer M.B. (1984). Effects of group identity on resource use in a simulated commons dilemma. J. Pers. Soc. Psychol..

[40] Allscheid S.P., Cellar D.F. (1996). An interactive approach to work motivation: The effects of competition, rewards, and goal difficulty on task performance. J. Bus. Psychol..

[41] Hogg M.A., Terry D.I., Terry D.J. (2000). Social Identity and Self-Categorization Processes in Organizational Contexts. Acad. Manag. Rev..

[42] Dick R., Wagner U., Stellmacher J., Christ O. (2005). Category salience and organizational identification. J. Occup. Organ. Psychol..

[43] Elliot A.J., Sheldon K.M. (1997). Avoidance achievement motivation: A personal goals analysis. J. Pers. Soc. Psychol..

[44] Hirst G., Van Knippenberg D., Zhou J. (2009). A Cross-Level Perspective on Employee Creativity: Goal Orientation, Team Learning Behavior, and Individual Creativity. Acad. Manag. J..

[45] Linnenbrink E.A., Pintrich P.R. (2002). Achievement Goal Theory and Affect: An Asymmetrical Bidirectional Model. Educ. Psychol..

[46] De Dreu C.K., Nijstad B.A., van Knippenberg D. (2008). Motivated information processing in group judgment and decision making. Pers. Soc. Psychol. Rev..

[47] Bunderson J.S., Sutcliffe K.M. (2003). Management team learning orientation and business unit performance. J. Appl. Psychol..

[48] Gong Y., Fan J. (2006). Longitudinal examination of the role of goal orientation in cross-cultural adjustment. J. Appl. Psychol..

[49] Payne S.C., Youngcourt S.S., Beaubien J.M. (2007). A meta-analytic examination of the goal orientation nomological net. J. Appl. Psychol..

[50] Vandewalle D. (1997). Development and Validation of a Work Domain Goal Orientation Instrument. Educ. Psychol. Meas..

[51] Dietz B., Van Knippenberg D., Hirst G., Restubog S.L.D. (2015). Outperforming Whom? A Multilevel Study of Performance-Prove Goal Orientation, Performance, and the Moderating Role of Shared Team Identification. J. Appl. Psychol..

[52] Lovelace K., Shapiro D.L., Weingart L.R. (2001). Maximizing cross-functional new product teams’ innovativeness and constraint adherence: A conflict communications perspective. Acad. Manag. J..

[53] Brislin R.W., Lonner W.J., Berry J.W. (1986). The wording and translation of research instruments. Field Methods in Cross-Cultural Research.

[54] Campion M.A., Medsker G.J., Higgs A.C. (1993). Relations between work group characteristics and effectiveness: Implications for designing effective work groups. Pers. Psychol..

[55] Bliese P.D. (1998). Team size, ICC values, team-level correlations: A simulation. Organ. Res. Methods.

[56] Bliese P.D., Klein K., Kozlowski S.W.J. (2000). Within-group agreement, non-independence, and reliability: Implications for data aggregation and analysis. Multilevel Theory, Research, and Methods in Organizations.

[57] Kirkman B.L., Chen G., Farh J.-L., Chen Z.X., Lowe K.B. (2009). Individual Power Distance Orientation and Follower Reactions to Transformational Leaders: A Cross-Level, Cross-Cultural Examination. Acad. Manag. J..

[58] Klein K., Bliese P.D., Kozlowski S.W.J., Dansereau F., Gavin M.A., Hofmann D.A., James L.R., Yammarino F.J., Bligh M.C., Klein K., Kozlowski S.W.J. (2000). Multilevel analytical techniques: Commonalities, differences, and continuing questions. Multilevel Theory, Research, and Methods in Organizations.

[59] Wu J.B., Tsui A.S., Kinicki A.J. (2010). Consequences of Differentiated Leadership in Groups. Acad. Manag. J..

[60] Chen X.-P., Xie X., Chang S. (2011). Cooperative and Competitive Orientation among Chinese People: Scale Development and Validation. Manag. Organ. Rev..

[61] Colquitt J.A., Rodell J.B. (2011). Justice, Trust, and Trustworthiness: A Longitudinal Analysis Integrating Three Theoretical Perspectives. Acad. Manag. J..

[62] Açıkgöz A., Günsel A., Bayyurt N., Kuzey C. (2014). Team climate, team cognition, team intuition, and software quality: The moderating role of project complexity. Group Decis. Negot..

[63] Naim M.F., Lenka U., Carayannis E., Tsui E. (2017). Linking knowledge sharing, competency development, and affective commitment: Evidence from Indian Gen Y employees. J. Knowl. Manag..

[64] Yeo G.B., Neal A. (2004). A Multilevel Analysis of Effort, Practice, and Performance: Effects; of Ability, Conscientiousness, and Goal Orientation. J. Appl. Psychol..

[65] Raudenbush S.W., Bryk A.S. (2002). Hierarchical Linear Models.

[66] Preacher K.J., Selig J.P. (2012). Advantages of Monte Carlo Confidence Intervals for Indirect Effects. Commun. Methods Meas..

[67] Dong Y., Bartol K.M., Zhang Z.X., Li C. (2017). Enhancing employee creativity via individual skill development and team knowledge sharing: Influences of dual-focused transformational leadership. J. Organ. Behav..

[68] Chen G., Gully S.M., Eden D. (2001). Validation of a New General Self-Efficacy Scale. Organ. Res. Methods.

[69] Barrick M.R., Bradley B.H., Kristof-Brown A.L., Colbert A.E. (2007). The Moderating Role of Top Management Team Interdependence: Implications for Real Teams and Working Groups. Acad. Manag. J..

[70] Baer M., Vadera A.K., Leenders R.T.A.J., Oldham G.R. (2014). Intergroup Competition as a Double-Edged Sword: How Sex Composition Regulates the Effects of Competition on Group Creativity. Organ. Sci..

[71] Wittchen M., Krimmel A., Kohler M., Hertel G. (2013). The two sides of competition: Competition-induced effort and affect during intergroup versus interindividual competition. Br. J. Psychol..

[72] Bono J.E., McNamara G. (2011). From the editors: Publishing in AMJ—Part 2: Research design. Acad. Manag. J..

[73] Podsakoff P.M., MacKenzie S.B., Lee J.-Y., Podsakoff N.P. (2003). Common method biases in behavioral research: A critical review of the literature and recommended remedies. J. Appl. Psychol..

[74] Podsakoff P.M., MacKenzie S.B., Podsakoff N.P. (2012). Sources of Method Bias in Social Science Research and Recommendations on How to Control It. Annu. Rev. Psychol..

[75] Ancona D.G. (1990). Outward bound: strategic for team survival in an organization. Acad. Manag. J..

